# Supplementary material for: Epstein-Barr virus LMP1 manipulates the content and functions of extracellular vesicles to enhance metastatic potential of recipient cells
Source: PLoS Pathog. 2020 Dec 31;16(12):e1009023. doi: 10.1371/journal.ppat.1009023 (PMC7774862; doi:10.1371/journal.ppat.1009023)
Supplement: Attachment — Submitted filename: Reviewer Rebuttals DN 9_23_20.docx [file ppat.1009023.s013.docx]

We thank all the reviewers for their helpful comments and appreciation of the experiments described in the manuscript

Reviewer #1: There are however few concerns including:

1. Some of the significant pieces of data from the proteomics (Figure 2) need to be validate with WBs. This is to show full-length proteins and potential validation of some key proteins from Mass Spec.

We have now added Sup. Fig1C and together with Fig 8A, C they show different protein ID identified in our proteomics.

2. Data in Figure 3 is interesting, but does not correlate with the number of EVs to the number of the RNAs. The authors should consider using few miRNAs (or other RNAs) that could serve as control markers for copy number and then back calculate the number of the isolated RNAs (piRNA, miRNA, etc) to get a sense of their significance in downstream functional assays.

We thank the reviewer’s suggestion on miR quantification. Currently there is still debate on how many miRs are there per single EV. Chevillet et al showed that even for the most abundant miRs, there is less that one molecule in one hundred EV(1). Another recent publication using qPCR showed that there about 10 miR-1246 molecules per EV(2). Our current work does focus on miR quantification, but on which miR or group of miR may induce downstream function in the recipient cells. When we find the target miR, quantification assay will be one of the critical experiments we would like to do.

3. Some of the data in Figure 5A (WB) does not look convincing and needs to be redone.

We have now included western blots for figure 5A which show similar results that LMP1 modified EVs activate ERK/AKT pathways.

4. It is surprising not to use any mutants of LMP1 for specificity of function, especially for data in Figures 9 and 10.

We have now included Sup. Fig 2B which shows that HK1 cells expressing C-terminal activating region (CTAR) 1 enhances the cell attachment. This is in agreement with our previous publication showing that LMP1 increased EV secretion is mediated through CTAR1(3).

Reviewer #2: Please deposit raw RNAseq data with appropriate databases and provide accessions.

The data discussed in this publication have been deposited in NCBI's Gene Expression Omnibus and are accessible through GEO Series accession number GSE155202. (https://www.ncbi.nlm.nih.gov/geo/query/acc.cgi?acc=GSE155202)

Please include NTA acquisition and analysis details in the manuscript.

You may access and check the submission of experimental parameters to the EV-TRACK knowledgebase via the following URL: http://evtrack.org/review.php. Please use the EV-TRACK ID (EV200071) and the last name of the first author (Nkosi) to access our submission. We have also uploaded in the supplementary the NTA acquisition and analysis details

Reviewer #3: -Experiments in which LMP1 appears to modify MMP activity to promote cell invasion and cell attachment through integrins lack a positive control. This control is important to evaluate the significance of these changes that look moderate. In addition, comparisons with MCF10A cells that express LMP1 will allow for a better evaluation.

We thank the reviewer for this suggestion and proposed experiments. For positive control of the aforementioned experiments we have now data from HK1 cells expressing LMP1 and have run some comparative data analysis. For Fig 9 showing that LMP1 modified EVs enhance cell attachment through integrinα5, we have now added Sup. Fig 5 showing HK1 cells expressing LMP1 attachment in fibronectin coated surfaces and effect of integrinα5 neutralizing antibodies. Comparative analysis our data between HK1 cells expressing LMP1 and MCF10A cells incubated with LMP1 modified EVs showed that cell attachment in fibronectin coated is the same (Sup. Fig 5A-B). In cells, we also did not observe any effect of the integrinα5 neutralizing antibodies on cell attachment, similar results has been in observed in MCF10A cells expressing LMP1 (4). For invasion assays, we have included Sup. Fig 6 showing that HK1 cells expressing LMP1 show enhanced invasion capacity. We have also done comparative data analysis comparing the HK1 cells expressing LMP1 and MCF10A cells incubated with LMP1 modified EVs.

- Some dose dependent experiments are important to be included.

We have now added Sup. Fig 2D showing that cell attachment can be EV dose dependent

- It is unclear how many EVs have been used in most assays and for how long.

We agree with the reviewer and this has been added to in the methods and material section, see line (628-630) for xcelligence assays and see line (666-669) for RNA-seq. For cell attachment, proliferation migration and invasion assays, the MCF10A were mixed with EVs to a final concentration of 10ug/ml when seeded in the well. For RNA-seq, MCF10A cells were exposed to physiological concentrations of EVs (10E+12) every 24hrs before harvesting the cell lysates.

-It is unclear what is the percentage of EVs carrying LMP1.

We thank the reviewer for the suggestion this is an interesting question. While in our publications and others have been able to show that LMP1 enhance vesicle release (5-7), there is no data currently available showing percentage EVs carrying LMP1. Our data just shows that LMP1 is enriched in the vesicles release from cells expressing LMP1. With current technologies it is difficult to measure LMP1 at the single vesicle level to answer this question.

-Is there a change at the protein level of fibronectin in LMP1-expressing cells and in cells exposed to EVs carrying LMP1?

We have now added Figure 8E showing fibronectin, integrinα5, MMP12.MMP9 levels in MCF10A cells exposed to EVs

Part III – Minor Issues: Editorial and Data Presentation Modifications

Please use this section for editorial suggestions as well as relatively minor modifications of existing data that would enhance clarity.

Reviewer #1: None

Reviewer #2: For graphs showing data for which statistical comparisons are done: would prefer dots rather than bar graphs as better indication of variability.

The bar graphs have been updated as suggested.

Recommended for replication purposes: deposit EV methods with EV-TRACK (evtrack.org) and give the accession citing van Deun, et al, Nat Methods, 2017.

You may access and check the submission of experimental parameters to the EV-TRACK knowledgebase via the following URL: http://evtrack.org/review.php. Please use the EV-TRACK ID (EV200071) and the last name of the first author (Nkosi) to access our submission.

"Secrete" in my understanding means release from within the cell, e.g. through endosomal pathways. Unless that is the intent, would recommend "release" as more general.

We have amended as suggested.

Further editing for accuracy and concision is recommended. For example, "Pivotal to these purification methods is the ability to harvest EVs that retain all the biological properties EVs supposedly encompass," could be better rendered as "An ideal purification method would retain EV function(s)."

We have amended that sentence

Reviewer #3: -There is a mislabeling of the panels in Fig. 6

This has been corrected now.

**References**

1.	Chevillet JR, Kang Q, Ruf IK, Briggs HA, Vojtech LN, Hughes SM, et al. Quantitative and stoichiometric analysis of the microRNA content of exosomes. Proceedings of the National Academy of Sciences. 2014;111(41):14888.

2.	Stevanato L, Thanabalasundaram L, Vysokov N, Sinden JD. Investigation of Content, Stoichiometry and Transfer of miRNA from Human Neural Stem Cell Line Derived Exosomes. PLoS One. 2016;11(1):e0146353.

3.	Hurwitz SN, Cheerathodi MR, Nkosi D, York SB, Meckes DG. Tetraspanin CD63 bridges autophagic and endosomal processes to regulate exosomal secretion and intracellular signaling of Epstein-Barr virus LMP1. Journal of Virology. 2017.

4.	Wasil LR, Shair KHY. Epstein–Barr virus LMP1 induces focal adhesions and epithelial cell migration through effects on integrin-α5 and N-cadherin. Oncogenesis. 2015;4(10):e171.

5.	Hurwitz SN, Nkosi D, Conlon MM, York SB, Liu X, Tremblay DC, et al. CD63 Regulates Epstein-Barr Virus LMP1 Exosomal Packaging, Enhancement of Vesicle Production, and Noncanonical NF-κB Signaling. Journal of Virology. 2017;91(5):e02251-16.

6.	Nkosi D, Howell LA, Cheerathodi MR, Hurwitz SN, Tremblay DC, Liu X, et al. Transmembrane Domains Mediate Intra- and Extra-cellular Trafficking of Epstein-Barr Virus LMP1. Journal of Virology. 2018.

7.	Verweij FJ, Eijndhoven MAJv, Hopmans ES, Vendrig T, Wurdinger T, Cahir‐McFarland E, et al. LMP1 association with CD63 in endosomes and secretion via exosomes limits constitutive NF‐κB activation. 2011.
